# Burden of Idiopathic Pulmonary Fibrosis Progression: A 5-Year Longitudinal Follow-Up Study

**DOI:** 10.1371/journal.pone.0166462

**Published:** 2017-01-18

**Authors:** Vincent Cottin, Aurélie Schmidt, Laura Catella, Fanny Porte, Céline Fernandez-Montoya, Katell Le Lay, Stève Bénard

**Affiliations:** 1 National Reference Center of rare pulmonary diseases, Department of Respiratory Medicine, Groupement Hospitalier Est-Hôpital Louis Pradel, University Claude Bernard Lyon 1, Lyon, France; 2 stève consultants, Oullins, France; 3 Boehringer Ingelheim, Paris, France; Helmholtz Zentrum München, GERMANY

## Abstract

Idiopathic pulmonary fibrosis (IPF) is a fatal lung disease with an unpredictable course. An observational study was set up using the French hospital discharge database to describe the reasons, outcomes and costs of hospitalisations related to this disease. Patients newly hospitalised for idiopathic pulmonary fibrosis (ICD-10 code: J84.1) in 2008 were identified and followed for 5 years. As J84.1 includes other fibrotic pulmonary diseases, an algorithm excluding age<50 years and presence of a differential diagnosis in the following year was defined. Overall, 6,476 patients were identified; of whom 30% were admitted through the emergency unit and 12% died during their first hospitalisation. Most of patients were hospitalised at least once for one or several acute events (n = 5,635; 87.0% of patients), of whom 36.5% of patients with an acute respiratory worsening (in-hospital mortality of 17.0% and median cost of €3,224; interquartile range (IQR €889–6,092)), 43.7% of patients with a respiratory infection (in-hospital mortality of 29.5% and median cost of €5,432 (IQR, €3,620–9,115)) and 51.7% of patients with a cardiac event (in-hospital mortality of 35.7% and median cost of €4,584 (IQR, €2,803–6,399)); 30.2% of these events occurred during the first hospitalisation. Finally, the 3-year in-hospital mortality crude rate was 36.8%. This study is the first providing extensive data on hospitalisations in patients with pulmonary fibrosis, mostly idiopathic, in France, demonstrating high burden and hospital cost.

## Introduction

Idiopathic pulmonary fibrosis (IPF) is a specific form of chronic, progressive, fibrosing interstitial pneumonia of unknown cause and limited to the lungs. It is a rare disease with an incidence estimated at 3.81 per 100,000 inhabitants in Europe [1].

According to an international consensus statement jointly issued by the American Thoracic Society, the European Respiratory Society, the Japanese Respiratory Society and the Latin American Thoracic Association, IPF is a distinct clinical entity associated with the histological and/or radiological appearance of usual interstitial pneumonia [2]. The definition of IPF requires the exclusion of other forms of interstitial pneumonia including other idiopathic interstitial pneumonias and interstitial lung diseases associated with environmental exposure, medication or systemic disease. IPF predominantly presents in individuals older than 50 years [2,3] with a preponderance in men and previous or current smokers. Patients present with chronic exertional dyspnoea, bibasilar inspiratory crackles, and commonly with cough, and finger clubbing.

IPF is characterised by progressive worsening of dyspnoea and lung function. This chronic disease is associated with a poor prognosis [2,4]. The median survival of patients diagnosed with this condition is estimated between 3 and 5 years [5]. The natural disease course is variable and characterised by various acute events, of whom idiopathic acute exacerbations are a major concern [6,7]. Currently there are no markers to reliably predict the clinical course in an individual patient. Little is known about the incidence and outcomes after acute events in Europe. While studies with small samples have focused on the poor prognosis of patients with severe IPF admitted to intensive care units in Europe and in the United States (US) [8–12], only one recent single-centre US study has evaluated the reasons for hospital admission and the outcomes for patients with IPF [13]. The results showed that hospitalisations, especially those related to respiratory problems, were common, and were associated with high in-hospital mortality.

In addition to the lack of European studies on IPF hospitalisations, data related to the cost of this disease are scarce. Though, we know that the management of IPF is resource-intensive given the difficulty to confirm the diagnosis and the multiple related comorbidities and complications. In order to optimise the patient care management to be as efficient as possible on tight budgets, it is important to understand the burden of this disease. Furthermore, the introduction of new pharmacologic treatments can lead to change the management of patients and the health-economic assessment of their incremental cost-effectiveness ratio is key to help decision-makers.

We therefore set up a 5-year population-level longitudinal study, based on data from the exhaustive French hospital discharge database to describe the reasons, outcomes and costs of hospitalisations in IPF patients.

## Material and Methods

### Data source

Subjects were identified from the French exhaustive national hospital discharge database (*Programme de Médicalisation des Systèmes d’Information* [PMSI]), which covers all stays in French public and private hospitals involved in medicine, surgery and obstetrics. In 2004, French hospitals adopted a prospective payment system. Since then, the PMSI database has become the basis of hospital funding by third-party payers [14]. A standard discharge summary report is generated for each hospital stay, and includes information on the patient characteristics (e.g. sex, age, residence code), main diagnosis that led to hospital admission and examinations carried out during hospitalisation. Diagnoses are coded using the International Classification of Diseases, 10^th^ revision (ICD-10) either as main diagnosis (one per stay), related diagnosis (one per stay) or significant associated diagnosis (as many as necessary). The discharge summary is then linked to a diagnosis-related group, used for the classification of hospital stays and with which is associated a national tariff.

Permission to extract and use the PMSI data was obtained from the National Commission on Informatics and Liberty (CNIL).

### Study population

**Fig 1:**
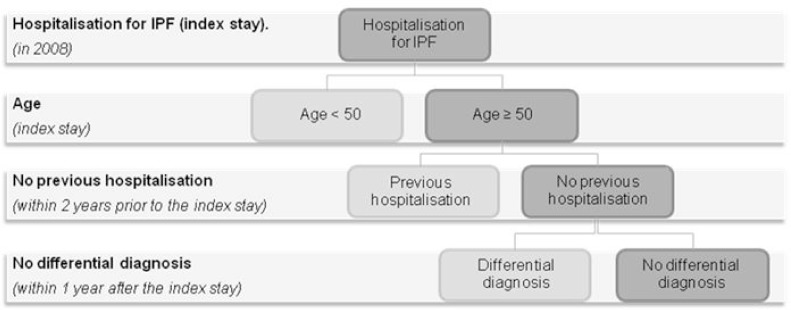
Patient’s flow chart. *Connective tissue diseases or pneumoconiosis ICD-10 codes: D86.*, J99.0, M05.1*, M30.*, M31.*, M32.*, M33.*, L94, M34.*, M35.*, J60, J920, J62.*, J63.*, J64, J65, J66.*, J67.*. See S1 Table for details.

An analysis of patients with IPF hospitalised in France (63.8 million inhabitants as of January 1^st^, 2008 [15]) was performed using the 2006–2013 PMSI database (i.e. the latest available data). Patients with a first admission related to IPF in 2008 were extracted using the existing ICD-10 code “J84.1: Other interstitial pulmonary diseases with fibrosis” as the main diagnosis, related diagnosis or significant associated diagnosis. In order to ascertain the first hospitalisation related to IPF, patients were excluded if they had been hospitalised for IPF in the 2 years before 2008. In addition, as the ICD-10 code J84.1 may be used for other very rare interstitial lung diseases with fibrosis, such as non-specific interstitial lung disease or cryptogenic organising pneumonia, and in order to increase specificity, patients were considered as having an IPF only if they were at least 50 years of age and had no differential diagnosis in the first year following the first hospitalisation related to IPF (i.e. for connective tissue diseases or pneumoconiosis, corresponding ICD-10 codes are described in S1 Table). One year of follow-up to identify differential diagnosis was considered as sufficient to detect a misdiagnosis (Fig 1). In addition, we performed a sensitivity analysis limited to patients with at least two hospitalisations related to IPF to assess the potential misdiagnosis of patients in the study population.

We selected a study period starting in 2008 and ending in 2013, as the median survival of patients with IPF is estimated to be between 3 and 5 years [5]. Thus, patients were followed until lung transplantation (considered as healed), death or the end of the 5-year follow-up period, whichever occurred first.

### Identification of hospitalisations

Hospitalisations analysed included:

- first hospitalisation identified as the first occurrence of an ICD-10 code J84.1,
- scheduled hospitalisation including monitoring visit and lung transplantation,
- acute events related to IPF leading to hospitalisations. They include respiratory infections (pneumonia, bronchitis, and influenza), pneumothorax, pulmonary embolism, myocardial infarction and cardiac events (heart failure, rhythm disorders or pulmonary hypertension). All of these acute events were identified using ICD-10 codes (S3 Table). In addition, we identified a sixth category of acute events; “unclassifiable respiratory events” (Table 1) assumed to be acute respiratory worsening without identified cause.

**Table 1:** Association of codes used to identify acute respiratory worsening (“unclassifiable respiratory events”).

Main diagnosis	Significant associated diagnosis
J84.1—Other interstitial pulmonary diseases with fibrosis	-
J96.0—Acute respiratory failure	J84.1—Other interstitial pulmonary diseases with fibrosis
-	J84.1—Other interstitial pulmonary diseases with fibrosis
	J96.0—Acute respiratory failure
J96.0—Acute respiratory failure	J96.1—Chronic respiratory failure
J96.0—Acute respiratory failure	J96.1+1- Restrictive chronic respiratory failure
J96.0—Acute respiratory failure	J96.9—Respiratory failure, unspecified
J96.1—Chronic respiratory failure	J96.0—Acute respiratory failure
J96.1+1—Restrictive chronic respiratory failure	J96.0—Acute respiratory failure
J96.9—Respiratory failure, unspecified	J96.0—Acute respiratory failure

We have not defined the main event of these acute hospitalisations; as a consequence, if a patient experienced two acute events during a stay, these two events were included in the results. Furthermore, acute events could have happened during the first hospitalisation.

### Collected data

Age gender and known comorbidities: chronic respiratory insufficiency, chronic obstructive pulmonary disease, heart failure, emphysema, pulmonary hypertension, lung cancer and sleep apnoea were collected at first hospitalisation.

For all hospitalisations, we analysed length of stay, admission to intensive care unit, admission to critical care unit, attendance in emergency department, use of ventilation and extracorporeal membrane oxygenation, management in expert centres (S4 Table) and in-hospital mortality.

### Statistical analysis

Patient socio-demographic characteristics and relevant comorbidities were analysed during the first hospitalisation. Data from the first hospitalisation and all subsequent hospitalisations were included in the clinical and economic analyses. The same method was used for the sensitivity analysis.

Quantitative data were expressed as median and interquartile range (IQR) and ordinal data were described by their numbers and percentages. The descriptive statistical analyses were performed using SAS V9.3^®^ software (SAS Institute Inc. Cary, NC, USA).

### Economic analysis

The complete cost of public and private hospitalisations has been estimated from the National Common Methodology Scale (ENCC). This provides a mean cost of hospitalisation according to the diagnosis-related group where the patient was classified as a function of his severity, comorbidities, and examinations, amongst other aspects. As 2013 ENCC data were not available at the time of this analysis, 2012 ENCC data were applied. Moreover, as 2008 ENCC data were missing for private hospitals [16], a 2007 ratio between public and private costs was estimated and applied to 2008 public costs. All costs are expressed in €2016 using the health service index [4011 E] (National Institute of Statistics Economical Studies [INSEE]).

## Results

### Characteristics of the study population

A total of 6,476 patients newly hospitalised for pulmonary fibrosis, mostly idiopathic, were identified in France in 2008. The median age was 77.0 (IQR, 69.0–83.0) years and 56.4% were men (Table 2). Pulmonary and respiratory comorbidities were common, as well as heart failure.

**Table 2:** Demographic characteristics and relevant comorbidities of patients at first hospitalisation.

Variable	Study population
	(n = 6,476)
**Men**	3,650 (56.4)
**Age, years**	77.0 (69.0–83.0)
**Relevant comorbidities**	
Chronic respiratory insufficiency	1,622 (25.0)
Chronic obstructive pulmonary disease	1,142 (17.6)
Heart failure	1,128 (17.4)
Emphysema	293 (4.5)
Pulmonary hypertension	290 (4.5)
Lung cancer	190 (2.9)
Sleep apnoea	155 (2.4)

Data are presented as n (%) or median (interquartile range).

## Hospitalisations

Over a 5-year maximum follow-up period, 21,660 hospitalisations (including the first hospitalisation) were extracted from the database, corresponding to a median of 2.0 (IQR, 1.0–4.0) hospital stays per patient. Median length of stay was 8.3 days (IQR, 4.3–14.0 days) and median cumulative length of stay was 20.0 days (IQR, 9.0–39.0 days) per patient. Among patients, 19.4% were hospitalised at least once in a reference/expert centre.

During the first hospitalisation, the median length of stay was 8.0 days (IQR, 2.0–15.0 days), with 85.3% lasting for more than 24 hours. These long lengths of stay were due largely to acute events that involved 4,729 patients (73.0%). The reasons of hospitalisation in the remaining patients were various: scheduled examination, related respiratory conditions (e.g. respiratory failure, chronic obstructive pulmonary disease, emphysema) or other diseases (e.g. cancer or coronary artery disease).

Over the 5-year maximum follow-up, the proportion of scheduled examinations was low, with 394 (6.1%) of patients hospitalised for this reason. Only 28 (0.4%) underwent lung transplantation. Besides 717 (11.1%) patients were receiving palliative care. Most of hospitalisations studied (16,106) were due to an acute event (Table 3), and concerned 5,635 patients (87.0%). The most frequent reasons for hospitalisation were cardiac events (51.7% of patients; heart failure (2,258 patients; 34.9%), rhythm disorders (2,183 patients; 33.7%) and pulmonary hypertension (736 patients; 11.4%), respiratory infections (43.7% of patients), and acute respiratory worsening (36.5% of patients). Pulmonary embolism occurred in 400 (7.8%) patients, myocardial infarction in 212 (3.3%) patients, and pneumothorax in 138 (2.1%) patients. All of these events were associated with high proportions of emergency admission or ventilation use (Table 3). Few hospitalisations led to intensive care unit (2.6%) or critical care unit (2.0%) or required extracorporeal membrane oxygenation (<0.1%). Overall 73.0% of patients were hospitalised for the first time due to acute events.

**Table 3:** Characteristics of hospitalisations for selected acute events.

	Respiratory related events			Cardiovascular events			Total
	Respiratory infection	Acute respiratory worsening	Pneumothorax	Cardiac events*	Pulmonary embolism	Myocardial infarction	
	(n = 4,639 stays)	(n = 3,865 stays)	(n = 174 stays)	(n = 9,135 stays)	(n = 534 stays)	(n = 271 stays)	(n = 16,106 stays)
**Length of stay, days**	10.0 (5.0–18.0)	4.0 (0.0–10.0)	13.0 (6.0–25.0)	8.0 (4.0–15.0)	9.5 (4.0–17.0)	9.0 (3.0–17.0)	7.0 (3.0–14.0)
**Occurrence during first hospitalisation**	1,366 (29.4)	1,838 (47.5)	69 (39.7)	2,004 (21.9)	179 (33.5)	79 (29.2)	4,729 (29.4)
**Length of first stay, days**	14.0 (8.0–24.0)	5.0 (0.0–11.0)	19.0 (11.0–31.0)	12.0 (7.0–20.0)	12.0 (6.0–24.0)	14.0 (8.0–26.0)	9.0 (4.0–17.0)
**Units**							
Emergency department	2,335 (50.3)	788 (20.4)	62 (35.6)	3,519 (38.5)	218 (40.8)	135 (49.8)	5,796 (36.0)
Intensive care unit	90 (1.9)	16 (0.4)	8 (4.6)	303 (3.3)	14 (2.6)	44 (16.2)	381 (1.8)
Critical care unit	143 (3.1)	35 (0.9)	10 (5.7)	182 (2.0)	14 (2.6)	6 (2.2)	881 (5.5)
**Non-invasive ventilation**	670 (14.4)	332 (8.6)	34 (19.5)	957 (10.5)	75 (14.0)	29 (10.7)	1,657 (10.3)
**Invasive ventilation**	492 (10.6)	112 (2.9)	45 (25.9)	482 (5.3)	49 (9.2)	30 (11.1)	881 (5.5)
**ECMO**	4 (0.1)	1 (<0.1)	2 (1.1)	2 (0.0)	1 (0.2)	0 (<0.1)	5 (<0.1)

Data are presented as n (%) or median (interquartile range). ECMO = ExtraCorporeal Membrane Oxygenation* Cardiac events include heart failure, rhythm disorders and pulmonary hypertension.

## In-Hospital Mortality

Overall, the cumulative in-hospital mortality increased from 12.8% during the first hospitalisation to 25.9% at 1 year, 36.8% at 3 years and 43.0% at 5 years. Among patients who experienced an acute event, the in-hospital mortality was 36.5% as shown in Table 4.

**Table 4:** Patients who experienced selected acute events and associated in-hospital mortality.

	Respiratory-related events			Cardiovascular events			Total
*n = 6*,*476 patients*	Respiratory infection	Acute respiratory worsening	Pneumothorax	Cardiac events*	Pulmonary embolism	Myocardial infarction	
Number of patients	2,830 (43.7)	2,365 (36.5)	138 (2.1)	3,349 (51.7)	400 (6.2)	212 (3.4)	5,635 (87,0)
Number of stays per patient	1.0 (1.0–2.0)	1.0 (1.0–2.0)	1.0 (1.0–1.0)	2.0 (1.0–3.0)	1.0 (1.0–1.0)	1.0 (1.0–1.0)	2.0 (1.0–3.0)
Associated in-hospital mortality	835 (29.5)	401 (17.0)	45 (32.6)	1,196 (35.7)	115 (28.8)	57 (26.9)	2,058 (36.5)

Data are presented as n (%) or median (interquartile range).

* Cardiac events include heart failure (2,258 patients; 34.9%), rhythm disorders (2,183 patients; 33.7%) and pulmonary hypertension (736 patients; 11.4%).

### Economic analysis

The median cost for the first hospitalisation was €4,510 (IQR, €1,978–6,860). Hospitalisations for the management of acute events had very high costs that are described in Table 5. Furthermore, the cost per stay for lung transplantation was €77,208 (IQR, €62,493–97,046) and the median cost of palliative care was €6,697 (IQR, €6,203–7,616).

**Table 5:** Median costs (€2016) of acute events in IPF patients.

	Median costs (IQR)
Pneumothorax	€6,513 (€4,395–12,015)
Pulmonary embolism	€6,198 (€4,058–8,826)
Myocardial infarction	€6,085 (€4,273–7,779)
Respiratory infection	€5,432 (€3,620–9,115)
Cardiac events*	€4,584 (€2,803–6,399)
Acute respiratory worsening	€3,224 (€889–6,092)

* Cardiac events include heart failure, rhythm disorders and pulmonary hypertension

### Sensitivity analysis

We identified 2,691 patients with at least 2 hospitalisations related to IPF (first hospitalisation in 2008). The median age was 76.0 (IQR, 69.0–82.0) years and 58.1% were men. Among patients, 24.7% were hospitalised at least once in a reference centre. Finally, the 3-year in-hospital mortality was 37.7%. Overall, by using a more restricted definition, including at least 2 hospitalisations related to IPF, characteristics of the population were similar; suggesting the impact of misdiagnosed patients is limited in the studied population.

## Discussion

This nationwide study shows that patients with pulmonary fibrosis, mostly idiopathic, are hospitalised for a variety of serious causes. The first hospitalisation was related to an acute event for nearly 75% of the population. When hospitalised, patients face a poor prognosis, with an in-hospital mortality of 12.8% during the first hospitalisation and 36.8% at 3 years. It should be noted that the PMSI database collects only secondary care data, which covers the mortality occurring during hospitalisations. As patients dying outside hospitals are not counted by the PMSI, the mortality, hereby reported, corresponds to the minimal estimate.

This study is the first to provide extensive data on the hospital management of patients with pulmonary fibrosis, mostly idiopathic, in France. Limited data are currently available to provide an overview of routine practice in patients with IPF. In a recent study based on data collected between 1997 and 2012 in the US, Brown et al. reported the incidence and outcomes of hospitalisations related to IPF. Although management in the US may differ from the one in Europe, some similarities with our findings are apparent [13]. Overall, they observed a mortality of 22.4% in a cohort of 97 IPF patients with a respiratory hospitalisation compare to an in-hospital mortality ranging from 17.0% to 32.6% for patients experiencing respiratory-related events in our study. Furthermore mean lengths of stay were similar (8.6 *vs* 6.9 days in our study). In 2013, Navaratnam et al. assessed IPF-related hospitalisations in England from 1998 to 2010 through the Hospital Episode Statistics database, but the analysis was limited to socio-demographic characteristics of patients (notably age and sex) and length and cost of stays [17]. They reported a length of stay of 7.2 days in the 1998–1999 calendar period that decreased to 5.1 days in the 2009–2010 calendar period.

Other studies involving patients with IPF followed in primary and secondary care settings reported 12% of idiopathic acute exacerbation at 1 year, with 37% of mortality after an idiopathic acute exacerbation [18]; overall, 78% of deaths were related to respiratory events. The proportion of hospitalisations in patients with IPF reported in the literature ranges from 25% to 50% [13,19].

Collard et al. [20] recently reported healthcare resource use and costs for patients with IPF (involving patients aged over 65 years) using Medicare data, the largest US payer covering the elderly. The study included 7,855 patients with IPF and showed similar results to our study. Patients hospitalised for IPF had a high risk of emergency room visits (39.6%). In our study, attendance in emergency departments ranged from 20.4% for acute respiratory worsening to 50.3% for respiratory infection. However, the study periods differ, as the study by Collard et al. included patients since diagnosis whereas we started to follow our patients after their first hospitalisation. Our study provides extensive data on hospital management for patients with IPF in France, which can be used for future economic analyses in France. Discharge databases permit estimations of the cost of a disease with a relatively high confidence, especially when few data are available, as for IPF. In this way, Collard et al. estimate a total medical cost per patient from the Medicare database, without further description by cost category [20]. Other studies have attempted to characterise the cost of IPF with less accuracy, using Delphi panel [21] or extrapolation [17].

Our study was based on a comprehensive national hospital database. However, this database was not developed for epidemiological purposes. Indeed, it is a tool built to measure hospital activity and to determine the cost of hospital stays according to the diagnosis-related group system. As the rate is related to the patients’ characteristics, clinical diagnoses and procedures, the collection of data over the hospital stay is accurate. A national agency (*Agence Technique de l'Information sur l'Hospitalisation*) manages the PMSI database and regularly publishes guidelines for coding at the hospital level under the supervision of a physician. This database is increasingly being used as a reference for epidemiological, medical and economic studies in France, including by health authorities. In oncology, for example, external validations of the PMSI data, performed in existing registries, showed high sensitivity and positive predictive values [22].

This study has some limitations. The ICD-10 code used to identify our population (“J84.1”) is not specific to IPF. It can be used for other very rare interstitial lung diseases with fibrosis. We partially overcame this limitation by limiting IPF cases to patients aged over 50 years (other diseases included are more likely to affect middle-aged people) and without a differential diagnoses (connective disease or pneumoconiosis) in the year following the first hospitalisation. Indeed, this period is assumed to be sufficient to detect a potential misdiagnosis. With this very careful approach, we believe the remaining small chance that some patients may have some interstitial lung diseases other than IPF is small and unlikely to affect the general findings of the study. Furthermore, in order to assess the impact of possible misclassification, we performed a sensitivity analysis limited to patients with at least two hospitalisations related to IPF and found consistent results in the patients’ characteristics. Moreover, the patients studied and presented in the manuscript have been hospitalised, which likely explains the older age and the balanced sex ratio compared to what is reported in cohort studies. Indeed, this population is slightly different from that of patients who are followed in clinical practice with less advanced disease and not experiencing severe acute events leading to acute deterioration. Other discharge database studies [17,23–26] have also been performed using the ICD-10 code “J84.1” or the ICD-9 code “516.3” with satisfying results. Although these 2 codes slightly differ (S1 Appendix), we can assume that the results are generally comparable as differences represent very rare diseases. In order to validate the accuracy of the ICD-9 code “516.3”, Agabiti et al. cross-checked their data against a chart review of 404 individual IPF cases collected from six Italian hospitals. They found no significant difference in the distribution of patient demographics and clinical characteristics between the subsample and the database population [23], thus validating the proper selection of IPF patients using this code. Very recently, a study presented another method to assess the performance of algorithms applied to identify IPF patients including validation by calculating positive predictive value using medical records [27]. This approach, which may be more accurate, could not be applied in our study, due to the difficulty of obtaining nominative patient-level data in France.

Presumably, acute respiratory worsening are likely to be acute exacerbation, notably idiopathic, however it cannot be verified in the absence of patient-level data. Besides, Moua et al. recently reported an extremely high mortality of 55%, in a cohort of 100 IPF patients hospitalised for acute respiratory worsening of whom 60% have a suspected acute exacerbation [28], suggesting that our population of patients experiencing acute respiratory worsening contains fewer acute exacerbations. As not all acute respiratory worsening are idiopathic acute exacerbation costs associated seem underestimated (€3,224). By assuming that acute exacerbation are managed as an “acute respiratory failure” (ICD-10 code J96.0) whatever its cause, we can approximate a median cost of €6,203.42 (IQR €533.58–104,789.35) per stay. Indeed, in our cohort, 1,505 (23.2%) patients were identified with at least one hospitalisation for an acute respiratory failure. Although the coding of J96.0 can be associated with different acute events and does not only represent idiopathic acute exacerbations, it emphasizes the fact that mixing idiopathic acute exacerbations of IPF with other acute respiratory worsening may underestimate the real burden of these events, which is probably in between. We consider that events of acute respiratory worsening in our study definitely represent severe events associated with significant mortality, regardless of their being genuine idiopathic acute exacerbations of IPF. Besides, in clinical practice, the diagnosis of idiopathic acute exacerbation related to IPF can be difficult, especially to rule out known causes of acute respiratory worsening, such as infection, aspiration, and air pollution, etc, which may have similar features and prognosis as idiopathic acute exacerbations. Specifically, these events are associated with high morbidity and mortality irrespective of etiology. A novel approach to the classification of acute respiratory exacerbation events has been recently proposed for patients with IPF, focusing on clinical and radiological findings consistent with an underlying process of acute worsening of parenchymal lung disease, and emphasizing that all acute events are severe and not only idiopathic acute exacerbations [7].

Providing an overview of the current practice regarding IPF-related hospitalisations and additional information on related disease management costs is essential in decision-making. Even if the study was performed between 2008 and 2013, before the publication in 2013 of French guidelines on the management of IPF [29], we believe that our results are still in line with current practices and may be used in the present context. They are useful to understand the care pathways and the main complications of IPF, in order to optimise the patient care management and ensure the best efficiency possible on tight budgets, notably by the publication of clinical guidelines. Moreover, the recent market authorization of two novel drug agent, pirfenidone and nintedanib, will obviously have an impact on IPF management costs as they have been proved to slow the rate of disease progression in IPF [30,31], hence, should lead to a diminution of health resources. Therefore, the results of our study may help to assess the cost-effectiveness of these drugs and thus to develop new pharmacologic strategy recommendations. The scope of evaluation being limited to outcomes and disease costs during hospitalisations, which represent the main drivers, future research would be needed to estimate the global impact of IPF in real-life practice, including primary care.

## Conclusion

Progression of IPF leads to hospitalisations, largely related to acute events, and is associated with a poor prognosis and high associated costs. Our study shows the substantial burden of pulmonary fibrosis, mostly idiopathic, in hospital settings, especially when patients experience acute respiratory events. The new data, this study provides, can be used to drive health-economic evaluation in order to help develop more accurate pharmacologic strategy guidelines.

## Supporting Information

S1 Appendix Definition of ICD-9 and ICD-10 codes including IPF. (DOCX)

S1 Table ICD-10 codes of differential diagnoses of IPF. (DOCX)

S2 Table ICD-10 codes of comorbidities studied. (DOCX)

S3 Table ICD-10 codes of acute events studied. (DOCX)

S4 Table Reference and competence (expert) centres of IPF in France. (DOCX)
