# Supplementary material for: Burden of Idiopathic Pulmonary Fibrosis Progression: A 5-Year Longitudinal Follow-Up Study
Source: PLoS One. 2017 Jan 18;12(1):e0166462. doi: 10.1371/journal.pone.0166462 (PMC5242514; doi:10.1371/journal.pone.0166462)
Supplement: S1 Appendix — (DOCX) [file pone.0166462.s001.docx]

# S1 Appendix. Definition of ICD-9 and ICD-10 codes including IPF.

# ICD-10 code J84.1 corresponds to Idiopathic fibrosing alveolitis and includes alveolar capillary block, diffuse (idiopathic) (interstitial) pulmonary fibrosis and Hamman-Rich syndrome.

**ICD-9 code 516.3** corresponds to Other interstitial pulmonary diseases with fibrosis and includes diffuse pulmonary fibrosis, fibrosing alveolitis (cryptogenic), Hamman-Rich syndrome, idiopathic pulmonary fibrosis and usual interstitial pneumonia.
