# Supplementary material for: Burden of Idiopathic Pulmonary Fibrosis Progression: A 5-Year Longitudinal Follow-Up Study
Source: PLoS One. 2017 Jan 18;12(1):e0166462. doi: 10.1371/journal.pone.0166462 (PMC5242514; doi:10.1371/journal.pone.0166462)
Supplement: S1 Table — (DOCX) [file pone.0166462.s002.docx]

S1 Table. ICD-10 codes of differential diagnoses of IPF.

| **Differential diagnosis** | **ICD-10 codes** | **Label** |
| --- | --- | --- |
| **Connective tissue diseases** | D86.* | Sarcoïdosis |
|  | J99.0, M05.1 | Rheumatoid Lung Disease With Rheumatoid Arthritis |
|  | M30.* | Polyarteritis nodosa and related conditions |
|  | M31.* | Other necrotizing vasculopathies |
|  | M32.* | Systemic lupus erythematosus |
|  | M33.* | Dermatopolymyositis |
|  | L94.*, M34.* | Scleroderma |
|  | M35.* | Other systemic involvement of connective tissue |
| Pneumoconiosis | J60 | Coalworker's pneumoconiosis |
|  | J61, J92.0 | Asbestosis / Pleural Plaque With Presence Of Asbestos |
|  | J62.* | Pneumoconiosis due to dust containing silica |
|  | J63.* | Pneumoconiosis due to other inorganic dusts |
|  | J64 | Unspecified pneumoconiosis |
|  | J65 | Pneumoconiosis associated with tuberculosis |
|  | J66.* | Airway disease due to specific organic dust |
|  | J67* | Hypersensitivity pneumonitis due to organic dust |

ICD=International Classification of Diseases
