# Supplementary material for: Burden of Idiopathic Pulmonary Fibrosis Progression: A 5-Year Longitudinal Follow-Up Study
Source: PLoS One. 2017 Jan 18;12(1):e0166462. doi: 10.1371/journal.pone.0166462 (PMC5242514; doi:10.1371/journal.pone.0166462)
Supplement: S2 Table — (DOCX) [file pone.0166462.s003.docx]

S2 Table. ICD-10 codes of comorbidities studied.

| **Comorbidity** | **ICD-10 codes** |
| --- | --- |
| Chronic obstructive pulmonary disease | J44.8 |
| Chronic respiratory failure | J96.1 |
| Sleep apnea | G47.3 |
| Emphysema | J43* |
| Heart failure | I50* |
| Pulmonary hypertension | I27.0, I27.2 |
| Malignant neoplasm of bronchus and lung | C34* |

ICD=International Classification of Diseases
