# Supplementary material for: Burden of Idiopathic Pulmonary Fibrosis Progression: A 5-Year Longitudinal Follow-Up Study
Source: PLoS One. 2017 Jan 18;12(1):e0166462. doi: 10.1371/journal.pone.0166462 (PMC5242514; doi:10.1371/journal.pone.0166462)
Supplement: S3 Table — (DOCX) [file pone.0166462.s004.docx]

S3 Table. Codes for hospitalisations; scheduled and acute events.

|  | **Acute event** | **ICD-10 codes** |
| --- | --- | --- |
| ICD-10 codes | Monitoring visit | Z09 |
|  | Respiratory infection | A481, B250, J09, J10.*, J11.*, J12.*, J13, J14, J15.*, J16.*, J17.*, J18.*, J20.*, J21.*, J22, J40 |
|  | Pneumothorax | J93.* |
|  | Cardiac event | I50.*, I47.*, I48, I49.*, I270, I272 |
|  | Pulmonary embolism | I26.* |
|  | Myocardial infarction | I21.*, I22.* |
|  | Idiopathic acute exacerbation | \| **Main diagnosis** \| **Significant associated diagnosis** \| \| --- \| --- \| \| J84.1 \| -- \| \| J96.0 \| J84.1 \| \| -- \| J84.1 + J96.0 \| \| J96.0 \| J96.1 \| \| J96.0 \| J96.9 \| \| J96.1 \| J96.0 \| \| J96.9 \| J96.0 \| |
| DRG codes | Lung transplantation | 27C04* |

ICD = International Classification of Diseases, DRG = Diagnosis Related Group
