# Supplementary material for: Burden of Idiopathic Pulmonary Fibrosis Progression: A 5-Year Longitudinal Follow-Up Study
Source: PLoS One. 2017 Jan 18;12(1):e0166462. doi: 10.1371/journal.pone.0166462 (PMC5242514; doi:10.1371/journal.pone.0166462)
Supplement: S4 Table — (DOCX) [file pone.0166462.s005.docx]

S4 Table: Reference and competence (expert) centres of idiopathic pulmonary fibrosis in France

| **FINESS number** | **Hospital** |
| --- | --- |
| 690784186 | CHU de Lyon-GH Est - Hôpital Louis Pradel |
| 750100273 | CHU Paris Est – Hôpital Ténon |
| 750803447 | CHU Paris Ouest – Hôpital Européen Georges-Pompidou |
| 750100232 | CHU Paris Nord-Val de Seine – Hôpital Xavier Bichat-Claude Bernard |
| 930100037 | CHU Paris Seine-Saint-Denis – Hôpital Avicenne |
| 250006954 | Centre CHU de Besançon – Hôpital Jean Minjoz |
| 350005179 | CHU de Rennes – Hôpital Pontchaillou |
| 670780055/670000025 | CHU de Strasbourg – Hôpital Civil |
| 130784234 | CHU de Marseille – Hôpital Sud Sainte-Marguerite |
| 590780193 | CHRU de Lille – Hôpital Albert Calmette |

(see http://www.maladies-pulmonaires-rares.fr/centre-competence/carte-centre-competence)
